# Validation of the Hungarian version of the SRI Questionnaire

**DOI:** 10.1186/s12890-020-1171-5

**Published:** 2020-05-07

**Authors:** Luca Valko, Szabolcs Baglyas, Laszlo Kunos, Attila Terray-Horvath, Andras Lorx, Janos Gal, Wolfram Windisch

**Affiliations:** 1 Department of Anesthesiology and Intensive Therapy, Semmelweis University, Ulloi ut 78/B, Budapest, 1082 Hungary; 2 Department of Pulmonology, Semmelweis University, Tomo utca 25-29, Budapest, 1083 Hungary; 3 Department of Neurology, Hungarian Army Medical Center, Robert Karoly korut 44, Budapest, 1134 Hungary; 4 Department of Pneumology, Cologne Merheim Hospital, Kliniken der Stadt Köln gGmbH, Witten/Herdecke University, Ostmerheimer Strasse 200, 51109 Cologne, Germany

**Keywords:** Home mechanical ventilation, Quality of life, Questionnaire validation

## Abstract

**Background:**

Home mechanical ventilation is a reliable treatment for patients suffering from chronic respiratory failure, improving survival and quality of life. Prevalence has been increasing worldwide as a result of evolving technical possibilities, telemedicine and improving national guidelines. Projects to establish a national guideline and registry for patients treated with home mechanical ventilation are currently under way in Hungary and our aim was to validate a quality of life questionnaire suited for evaluation and follow up in this specific patient group. The Severe Respiratory Insufficiency Questionnaire (SRI) is a quality of life tool designed to evaluate patients receiving home mechanical ventilation and has been validated both in patient groups receiving invasive and noninvasive ventilation.

**Methods:**

The Hungarian version of the SRI was created using the translation-backtranslation method, which was then tested for validity, viability and reliability in a cohort involving patients from three centers, receiving long-term home mechanical ventilation for chronic respiratory failure through an invasive or noninvasive interface. Patient data was collected (demographic data, lung function test, arterial blood gas, ventilation settings) and quality of life was measured with the previously validated SF-36 and newly created Hungarian SRI Questionnaires at two time points.

**Results:**

One hundred four patients receiving home mechanical ventilation were enrolled. The time to complete the SRI Questionnaire was 8.6 (±3.1) minutes, 69.2% questionnaires were self-administered. Exploratory factor analysis explained 73.8% of the variance of the questionnaire, but resulted in 13 scales. We found correlations between the SRI subscale scores to corresponding scales of the previously validated general quality of life survey SF-36. The Cronbach alpha coefficient was 0.928 for the Summary Scale of the SRI Questionnaire, proving high internal consistency. Reproducibility was high for most scales, resulting in a high overall correlation for the summary score (0.877, *p* < 0.001).

**Conclusions:**

The Hungarian version of the SRI Questionnaire is a viable, valid, reliable and reproduceable quality of life tool applicable for patients treated with home mechanical ventilation.

## Background

Home mechanical ventilation (HMV) is a potential long term therapeutic intervention for patients with different forms of chronic respiratory failure [1]. Prevalence has been growing worldwide with the aid of evolving technical possibilities, telemedicine and improved national guidelines. The prevalence in Hungary has been reported to be lower than in leading countries worldwide, although improved reimbursement has vitalized the field and has resulted in increased awareness and evolving care for chronic respiratory failure patients [2]. Projects to establish a national guideline and registry for patients treated with home mechanical ventilation are currently under way in the country, hence the need to develop a quality of life survey for this specific group of patients that can be administered for adequate evaluation and follow up.

The Severe Respiratory Insufficiency Questionnaire (SRI) is a condition-specific quality of life survey that has been developed specifically for chronic respiratory failure patients and was originally published in German [3]. It has been validated in patients treated with home mechanical ventilation both through noninvasive and invasive modes of ventilation and has been found to correlate with long-term outcome [4–6]. It has been translated to several languages and validated using the translation-back translation method [7–13].

The aim of the current project was to create and validate a Hungarian version of the SRI Questionnaire for further use in patients evaluated for home mechanical ventilation.

## Methods

### SRI Questionnaire

The SRI Questionnaire is a self-administered quality of life verification tool which has been found to have high psychometric properties. It consists of 49 items and a corresponding 5 level Likert scale. The items are analyzed and grouped into 7 different subscales (respiratory complaints: SRI-RC – eight items, physical functioning: SRI-PF – six items, attendant symptoms and sleep: SRI-AS – seven items, social relationships: SRI-SR - six items, anxiety: SRI-AX - five items, psychosocial well-being: SRI-WB - nine items, and social functioning: SRI-SF - eight items), all of which result in a score ranging from 0 to 100. The summary score (SRI-SS) is calculated from the mean of all subscales, resulting in a range from 0 to 100, with higher scores signaling higher quality of life.

### Translation-back translation method

The original German questionnaire was translated by two certified translators from German to Hungarian in December of 2017. A unified first version of the questionnaire was compiled by a group of experts in the field of pulmonology, critical care and mechanical ventilation, which was then backtranslated to German. The original author group verified the backtranslation based on equivalence for all questions and the Hungarian version was subsequently revised based on equivalence discrepancies. This revised second version was then tested in a pilot study in February of 2018 and was amended, based on difficulties in understanding, into the third version. The third version of the Hungarian SRI Questionnaire was then tested for validity, viability and reliability in a large cohort.

### Patients

Patients were recruited through the Semmelweis University Home Mechanical Ventilation Program, the Department of Pulmonolgy of Semmelweis University and the Department of Neurology of the Hungarian Army Medical Center from February 2018 to August 2019. Adult, stable chronic respiratory failure patients receiving home mechanical ventilation with an aid of a bi-level ventilator through invasive or noninvasive interface were eligible for the validation study. Exclusion criteria were inability to cooperate with the survey, less than 3 months of treatment or acute worsening of chronic respiratory condition within the previous month. All patients were informed about the process of the validation study and signed written informed consent forms before enrollment. The study was approved by the ethical committee of Semmelweis University (TUKEB 249/2017).

During the enrollment, demographic data [age, gender, body mass index (BMI), education level, employment status, smoking history, disease], clinical parameters (lung function test and arterial blood gas sample) and ventilation characteristics [duration of ventilation, daily ventilation use during the previous month, interface type, mean inspiratory positive airway pressure (IPAP), expiratory positive airway pressure (EPAP), frequency, inspiratory time/exspiratory time ratio (Ti/Tt), patient triggered breath ratio values during the previous month] were recorded. Arterial blood gas sampling was performed minimum 15 min after discontinuing ventilation and/or oxygen supplementation, unless patients were ventilator dependent and could not be disconnected for even short periods of time. Lung function tests were performed with the Piston PinkFlow meter (Piston Ltd., Budapest, Hungary). Patients were asked to self-administer the Hungarian SRI and the 36 Item Short Form (SF-36) Questionnaires and were aided by the investigator if self-administration was not possible. Time to complete questionnaires was measured. Patients were asked to retake the survey in their home 1 week after enrollment to verify reproducibility. The repeated questionnaires were collected through mail, through electronic mail or at subsequent ambulatory visits.

### Psychometric properties

Psychometric properties were verified based on the protocol previously published during the Spanish validation of the SRI Questionnaire [7].

Viability was studied by recording the time spent to complete the questionnaire, ability to self-administer the questionnaire and the missing item rate for the questionnaire. Validity was determined by exploratory factor analysis of the 49 items in the Hungarian SRI, subsequent confirmatory factor analysis of subscales and by comparing the corresponding scales of the Hungarian SRI with the Hungarian SF-36 already in use [14].

Reliability was determined by testing the internal consistency using the Cronbach alpha coefficient. A scale was deemed reliable if Cronbach alpha coefficient was greater than 0.7 and if its items correlated better with their own scale than items of the rest of the scales. Reproducibility was assessed by determining the correlation of the results of the two questionnaires submitted by the same patient at different time points.

### Statistical analysis

Data are represented as mean(±standard deviation) for quantitative variables and n(%) for qualitative variables. Groups were compared using the paired Student t-test. Intergroup differences were determined using the analysis of variance (ANOVA) test. Factor analysis was performed, after testing for sample adequacy, using the principal component method with a varimax rotation, using an eigenvalue > 1 for extraction and further verified by scree plot. Confirmatory factor analysis was performed on each of the six subscales defined by the original German methodology of the Scoring Guide of the SRI Questionnaire. Scale correlations were determined using Pearson correlation. Results were deemed statistically significant at *p* < 0.05. Statistical analysis was performed with the Statistical Package for Social Sciences (SPSS, Chicago, IL, USA) version 25.

## Results

### Backtranslated questionnaire

The backtranslated first version of the original questionnaire was rated as “totally equivalent” for 9 items and “similar” for 36 items by the original author group. Four items rated “doubtful” in the backtranslated version were subsequently discussed and revised if needed by the expert panel. The changes effected 1 of the items, the doubtfulness of the other 3 items were thought to be a consequence of backtranslation issues and were unchanged as per the decision of the expert panel. The pilot study of the second version resulted in no apparent difficulties of understanding by test subjects and was accepted as the third version (Supplementary Material 1).

### Descriptive statistics

The total number of patients recruited for the study was 104, all patients completed the study. Mean age was 54.5 (±16.2) years, 77 (74.0%) patients were male. Highest level of education was primary school for 22 (21.1%), secondary school for 53 (51.0%), university for 29 (27.9%) patients. Current state of employment was employed for 27 (26.0%), never worked for 9 (8.6%), disabled/unable to work for 31 (29.8%) and pensioner for 37 (35.6%) patients. None of the patients were smoking at the time of enrollment, 47 (45.2%) never smoked, while 57 (54.8%) were prior smokers. Cumulative smoking was found to be 14.7 (±23.1) packyears/person. Cause for chronic respiratory failure and mechanical ventilation need was found to be chronic obstructive pulmonary disease (COPD) in 20 (19.2%), restrictive chest wall disease (RCWD) in 6 (5.8%), obstructive sleep apnea or obesity hypoventilation syndrome (OSA/OHS) in 45 (43.3%) and neuromuscular disease (NMD) in 31 (29.8%) patients, while two patients (1.9%) suffered from other causes (pulmonary fibrosis and scleroderma). The most frequently used interface was a full-face mask in 59 (56.7%) patients, while 23 (22.1%) used a total face mask, 8 (7.7%) used a nasal mask and 14 (13.5%) were ventilated through a tracheostomy. Patients received HMV for a mean of 9 (±4.8) hours per day, and they had been using HMV for 26.2 (±32.7) months. O_2_ supplementation was used in 46 (44.2%) patients, overall oxygen use was 1.2(±1.8) L/min/patient.

The clinical characteristics of the patient sample are summarized in Table 1.

**Table 1:** Clinical characteristics of the study population

	COPD	RCWD	OHS/OSAS	NMD	Total
n (%)	20(19.2%)	6(5.8%)	45(43.3%)	31(29.8%)	104(100%)
Age (yr)	66.0(±6.8)	36.5 ± 13.0)	57.3(±13.3)	46.7(±18.8)	54.5(±16.2)
Male (%)	15(75%)	1(8.3%)	35(77.8%)	7(88.4%)	77(74.0%)
BMI (kg/m^2^)	33.9(±7.4)	19.4(±3.4)	41.2(±10.8)	24.1(±4.4)	33.1(±11.6)
Smoking (packyear)	26.7(±29.6)	7.5(±17.9)	16.8(±22.3)	6.3(±16.9)	14.7(±23.1)
HMV (hr/d)	8.0(±2.0)	10.7(±6.4)	6.4(±1.7)	12.4(±5.7)	9.0(±4.8)
HMV (mo)	28.1(±29.6)	27.5(±39.6)	30.6(±37.2)	19.7(±26.8)	26.2(±32.7)
O_2_ need	14(70%)	4(6%)	19(42.2%)	7(22.6%)	46(44.2%)
FVC%	71.2(±23.6)	23.8(±12.5)	80.9 ± 19.6	36.1(±19.5	68.3(±28.9)
FEV_1_%	44.7(23.6)	23.8(±12.5)	72.5(±20.6)	36.4(±22.1)	53.3(±27.5)
FEV_1_/FVC%	63.0(±20.6)	86.3(±17.8)	92.2(±13.7)	100.5(±17.8)	88.0(±21.4)
PEF%	44.7(±23.7)	28.8(±16.3)	71.2(±24.4)	31.6(±21.0)	51.6(±28.9)
pH	7.40(±0.05)	7.38(±0.03)	7.41(±0.04)	7.40(±0.04)	7.40(±0.04)
pCO_2_ (mmHg)	44.8(±10.4)	43.8(±9.4)	42.3(±9.6)	41.4(±8.8)	42.6(±9.5)
pO_2_ (mmHg)	70.2(±15.6)	84.0(±10.3)	73.3(±10.4)	87.0(±10.6)	77.5(±13.3)
IPAP (cmH_2_O)	17.9(±3.3)	22.0(±3.2)	19.3(±4.7)	18.5(±3.9)	18.9(±4.1)
EPAP (cmH_2_O)	7.8(±2.9)	6.7(±2.9)	9.6(±3.1)	7.6(±3.4)	8.4(±3.3)
PTB (%)	62.7(±34.2)	25.5(±25.4)	65.5(±32.0)	45.9(±28.3)	57.0(±32.5)
VTE (mL)	669.1(±155.9)	367.9(±62.5)	648.3(±245.2)	483.6(±94.4)	585.5(±205.1)
T_i_/T_t_	31.7(±5.6)	32.0(±7.4)	36.4(±5.4)	32.1(±5.5)	33.8(±5.9)

*COPD* chronic obstructive pulmonary disease, *RCWD* restrictive chest wall disease, *OHS/OSAS* obesity hypoventilation syndrome/obstructive sleep apnea syndrome, *NMD* neuromuscular disease, *BMI* body mass index, *HMV* home mechanical ventilation, *FVC* forced vital capacity, *FEV1* forced expiratory volume within 1 s, *PEF* peak expiratory flow, *pCO2* partial CO2 pressure, *pO2* partial O2 pressure, *IPAP* inspiratory positive airway pressure, *EPAP* expiratory positive airway pressure, *PTB* patient triggered breath, *VTE* expiratory tidal volume, *Ti/Tt* inspiratory – total time ratio

**Fig. 1:**
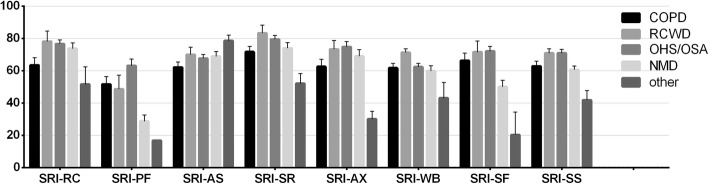
Box plot of SRI subscales in different diagnostic groups. Boxes represent means, whiskers represent standard error. COPD – chronic obstructive pulmonary disease, RCWD – restrictive chest wall disease, OHS/OSAS – obesity hypoventilation syndrome/obstructive sleep apnea syndrome, NMD – neuromuscular disease, SRI-RC - respiratory complaints, SRI-PF - physical functioning, SRI-AS - attendant symptoms and sleep, SRI-SR - social relationships, SRI-AX - anxiety, SRI-WB - psychosocial well-being, SRI-SF - social functioning, SRI-SS - summary scale

Quality of life scores were not homogenously distributed within patient groups (Fig. 1). SRI-RC, −PF, −AX, −SF and -SS scales showed notable differences in patient groups (*p* = 0.048, *p* < 0.001, *p* = 0.022, *p* < 0.001 and *p* = 0.003 respectively).

SRI-PF was diminished in NMD patients compared to COPD and OHS/OSAS patients (28.7 ± 21.9 compared to 51.6 ± 21.6 and 63.1 ± 27.4; *p* = 0.004 and *p* < 0.001 respectively). SRI-SF was less in RCDW and NMD patients than in OHS/OSAS patients (48.6 ± 21.0 and 50.0 ± 22.7 compared to 72.0 ± 20.4, *p* = 0.039 and *p* < 0.001 respectively). SRI-SS was less in NMD patients compared to OSAS/OHS patients (60.6 ± 13.2 compared to 70.8 ± 15.7, *p* = 0.033).

**Fig. 2:**
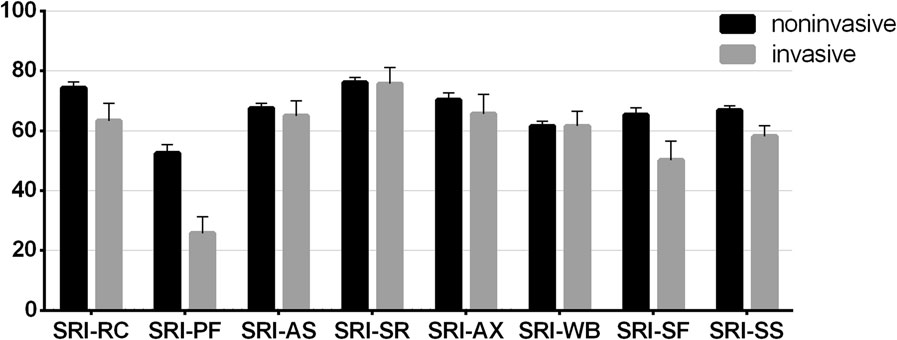
Boxplot of subscales in patients according to interface. Boxes represent means, whiskers represent standard error. SRI-RC - respiratory complaints, SRI-PF - physical functioning, SRI-AS - attendant symptoms and sleep, SRI-SR - social relationships, SRI-AX - anxiety, SRI-WB - psychosocial well-being, SRI-SF - social functioning, SRI-SS - summary scale

When comparing patients ventilated through noninvasive and invasive interface, we found that SRI-RC, −PF, SF and -SS subscale scores were higher in the noninvasive group (SRI-RC: 74.3 (±18.5) vs. 63.4 (±22.2), *p* = 0.049; SRI-PF: 52.5 (±27.7) vs. 25.7 (±20.5), *p* = 0.001; SRI-SF: 65.3 (±23.0) vs. 50.2 (±23.6), *p* = 0.025, SRI-SS: 66.8 (±15.1) vs. 58.2 (±13.6), *p* = 0.046), while other subscales showed no significant difference (see Fig. 2.).

### Viability

The time to complete the SRI was the same as the time for the SF-36: 8.6 (±3.1) minutes vs. 8.5 (±3.0); *p* = 0.587. 72 (69.2%) questionnaires were self-administered. Self administered questionnaires were completed faster (8.1 ± 2.8 vs. 9.7 ± 3.6, *p* = 0.019), diseases type and education level significantly influenced the time spent on the questionnaire (p = 0.025 and *p* = 0.002 respectively), but age of the patient did not correlate with the time to complete the SRI Questionnaire (correlation factor: − 0.006, *p* = 0.954). The reasons given for not self-administering the questionnaire were physically unable for 24 (23.1%) or eyesight problems for 11 (10.6%) patients. Overall missing items were 0.2 (±0.6) out of the 49 items for the SRI and 0.2 (±0.8) out of the 36 items for the SF-36. All questions were answered by 96–100% of patients. The question skipped most frequently for the SRI Questionnaire was question 31 (regarding the effect of the disease on relationships) by 4 (3.8%) patients, because they were not in a relationship.

### Validity

**Fig. 3:**
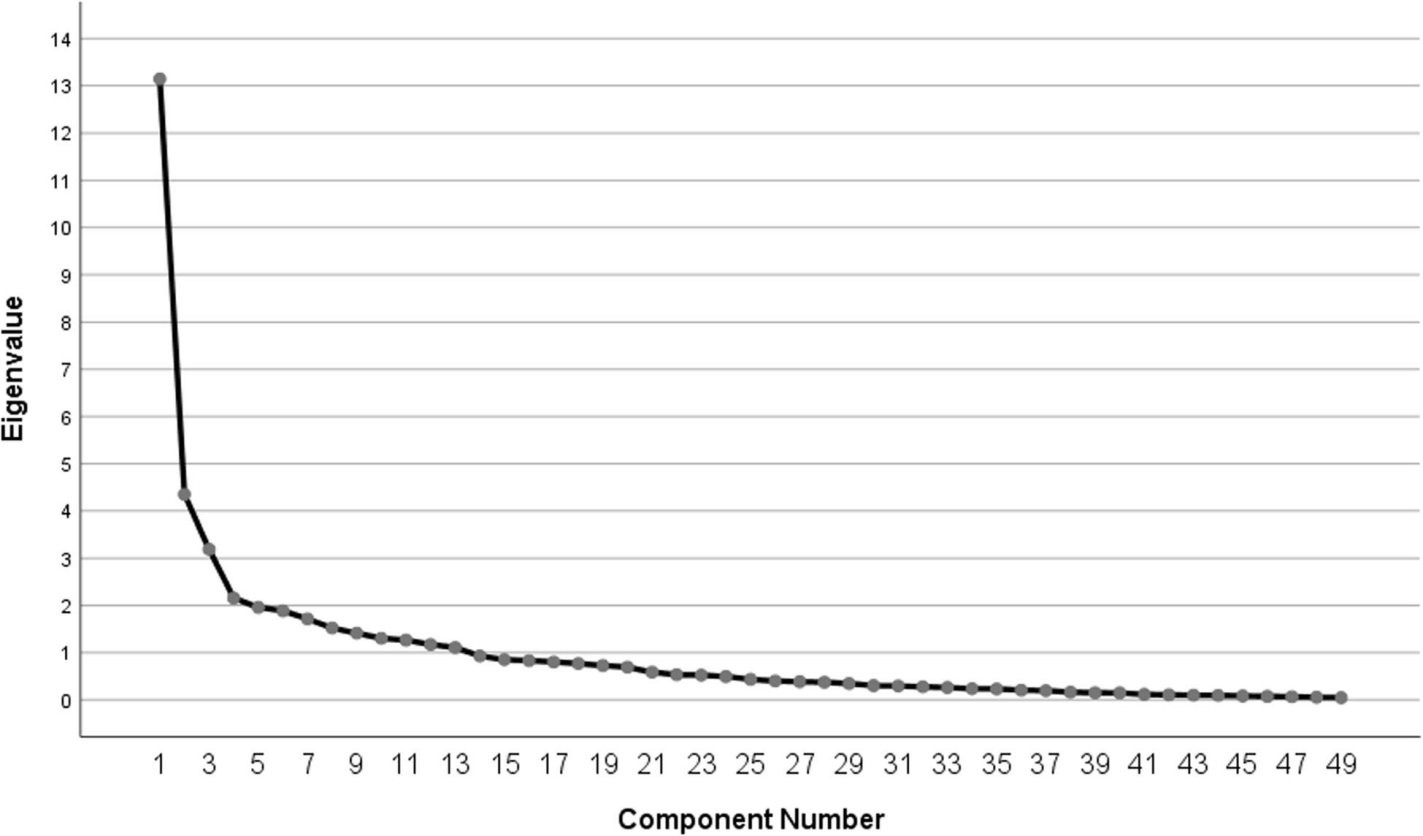
Scree plot for the exploratory factor analysis

Exploratory factor analysis explained 73.8% of the variance of the questionnaire, but it resulted in 13 scales, which is similar to results published by the Spanish validation of the SRI Questionnaire. Figure 3 shows the scree plot of the exploratory factor analysis. The slope of the curve significantly levels out after 4 components, but is further reduced below an eigenvalue of 1 by 9 subsequent components.

Conformatory factor analysis for the 7 subscales, defined by the original German version of the questionnare, showed one component for one, two components for five and three components for one of the subscales. The PF scale was the most retained, its factor analysis showing only one component. All other scales tended to be divided into two further scales, albeit showed significant correlations with each other. The RC’s first scale included items related to shortness of breath while the second to the ability to cough and expectorate (correlation factor: 0.466, *p* < 0.001). The AS’s two scales focused on general secondary symptoms and the ability to sleep through the night respectively (correlation factor: 0.257 *p* = 0.008). The SR’s first scale related to items concerning relationships to others, while the second focused on feelings of loneliness (correlation factor: 0.210, *p* = 0.032). The AX was also divided into two scales with some questions focusing on disease related anxieties and others related to social anxiety (correlation factor: 0.419, *p* < 0.001). The SF’s first scale focused on activities and leisure while the other one on social interactions (correlation factor: 0.832, *p* < 0.001). The WB was the most divided scale, with three components, the largest one with items evaluating depression while the other two related to feelings of frustration with the disease. All these also showed significant correlations (0.328 *p* = 0.001; 0.299 *p* = 0.002; 0.222 *p* = 0.024). The results of the factor analysis of the Hungarian version of the SRI Questionnaire are similar to other validation studies published previously (see Supplementary Material 3).

The correlation matrix for the SRI and SF-36 questionnaires is presented in Table 2.

**Table 2:** Correlation matrix: SRI and SF-36

	SF36-PF	SF36-PH	SF36-EP	SF36-E/F	SF36-EWB	SF36-SF	SF36-P	SF36-GH
SRI-RC	0.384**	0.499**	0.368**	0.523**	0.341**	0.397**	0.482**	0.591**
SRI-PF	0.842**	0.437**	0.304*	0.432**	0.317*	0.409**	0.277*	0.3434**
SRI-AS	0.290*	0.425**	0.385**	0.463**	0.432**	0.355**	0.557**	0.474**
SRI-SR	0.401**	0.390**	0.456**	0.552**	0.596**	0.537**	0.369**	0.378**
SRI-AX	0.418**	0.458**	0.340**	0.398**	0.342**	0.409**	0.330*	0.526**
SRI-WB	0.315*	0.353**	0.363**	0.611**	0.721**	0.591**	0.420**	0.514**
SRI-SF	0.732**	0.485**	0.428**	0.561**	0.473**	0.593**	0.385**	0.535**
SRI-SS	0.699**	0.590**	0.499**	0.667**	0.590**	0.626**	0.522**	0.660**

Values with *p* < 0.05 are marked with *. Values with *p* < 0.001 are marked with **. Strong correlations are marked with bold text. Abbreviations for the SRI scales: respiratory complaints (SRI-RC), physical functioning (SRI-PF), attendant symptoms and sleep (SRI-AS), social relationships (SRI-SR), anxiety (SRI-AX), psychosocial well-being (SRI-WB), social functioning (SRI-SF), summary scale (SRI-SS). Abbreviations for the SF-36 scales: physical functioning (SF36-PF), role limitations due to physical health (SF36-PH), role limitations due to emotional problems (SF36-EP), energy/fatigue (SF36-E/F), emotional well-being (SF36-EWB), social functioning (SF36-SF), pain (SF36-P) and general health (SF36-GH)

The best correlations of the two questionnaires were achieved between both PF scales (R: 0.842 *p* < 0.001). Other strong correlations were found between the SRI-SF and SF36-PF (0.732, *p* < 0.001), the SRI-SS and SF36-PF (0.699, *p* < 0.001) and the SRI-WB and SF36-EBW scales (0.721, *p* < 0.001). Moderately strong correlations were found between the SRI-WB and SF36-E/F (0.611, *p* < 0.001), the SRI-SS and SF36-E/F (0.667, *p* < 0.001), the SRI-SS and SF36-SF (0.626), *p* < 0.001) and SRI-SS and SF36-GH scales (0.660, *p* < 0.001).

### Reliability

The Cronbach alpha coefficient was 0.928 for the summary scale of the Hungarian SRI Questionnaire. The Cronbach alpha coefficients, and correlation of each item with its own scale correcting for overlap and with the rest of the scales are listed in Table 3.

**Table 3:** Cronbach’s alpha reliability

Scale	Cronbach alpha	ICQ (min-max)	ICS (min-max)
SRI-RC	0.810	−0.053-0.621	0.758–0.866
SRI-PF	0.836	−0.382-0.704	0.781–0.880
SRI-AS	0.635	−0.330-0.584	0.516–0.734
SRI-SR	0.612	−0.350-0.620	0.484–0.717
SRI-AX	0.726	−0.451-0.569	0.633–0.802
SRI-WB	0.630	−0.492-0.591	0.513–0.728
SRI-SF	0.849	−0.452-0.704	0.800–0.890

*ICQ* item correlation coefficient with the rest of the questionnaire, *ICS* item correlation coefficient with its own scale, *SRI-RC* respiratory complaints, *SRI-PF* physical functioning, *SRI-AS* attendant symptoms and sleep, *SRI-SR* social relationships, *SRI-AX* anxiety, *SRI-WB* psychosocial well-being, *SRI-SF* social functioning, SRI-SS - summary scale

The Cronbach alpha coefficient was above 0.7 for most scales except AS, SR and WB, proving similar reliability to previously translated questionnaires (see Supplementary Material 3). Correlations of an item to its own scale, correcting for overlap (item correlation coefficient with its own scale [ICS]), were good compared with the correlations with the rest of the scales in the questionnaire (item correlation coefficient with the rest of the questionnaire [ICQ]), reaching higher correlations in ICS than in ICQ.

### Reproducibility

Correlations between the results of the two SRI Questionnaires submitted at different time points are listed in Table 4.

**Table 4:** Correlation of repeated punctuation of the SRI Questionnaire

Scale	Correlation factor	***p***-value
SRI-RC	0.778	*p* < 0.001
SRI-PF	0.859	*p* < 0.001
SRI-AS	0.820	*p* < 0.001
SRI-SR	0.676	*p* < 0.001
SRI-AX	0.774	*p* < 0.001
SRI-WB	0.736	*p* < 0.001
SRI-SF	0.823	*p* < 0.001
SRI-SS	0.877	*p* < 0.001

*SRI-RC* respiratory complaints, *SRI-PF* physical functioning, *SRI-AS* attendant symptoms and sleep, *SRI-SR* social relationships, *SRI-AX* anxiety, *SRI-WB* psychosocial well-being, *SRI-SF* social functioning, *SRI-SS* summary scale

Reproducibility was high for most scales, resulting in a high overall correlation for the summary score (0.877, *p* < 0.001).

## Discussion

The SRI Questionnaire is a quality of life tool designed to evaluate chronic respiratory failure patients and has high psychometric properties. Our goal was the transcultural adaptation of the questionnaire through the translation-back translation method involving the original author group. The results of the current validation study prove that the Hungarian version of the questionnaire has psychometric properties similar the original version and its subsequent translations and is suitable for assessing quality of life in patients requiring home mechanical ventilation.

Our study population included patients with common diagnoses that are currently advised to be treated through home mechanical ventilation [15–17] with the characteristics of patients similar to the original study group [3], although there are also notable differences in the patient composition. The most common diagnosis for home mechanical ventilation in our study population was OHS/OSAS, which is an increasingly common indication for HMV in recent years [18]. It has been reported before that the majority of Hungary’s HMV population has begun treatment in the last 5 years with OHS being the most frequent indication, attributing to the composition of our cohort [2]. Our results of the SRI Questionnaire in this patient group indicate that OHS/OSAS causes similair quality of life limitation to other chronic respiratory failure conditions, with SRI-PF and -WB scales being the most affected aspects.

The second most frequent diagnosis in our cohort was NMD, while COPD and RCWD were less common. It should be noted that until recently, COPD diagnosis alone was not sufficient to apply for reimbursement in the Hungarian health care system, so most patients are predictably overlap patients (COPD and OSAS) as indicated by the relatively high FEV1/FVC% values of this patient group. The relatively normal pCO_2_ values of this patient group are notable and reflect successful achievement of treatment goals (as the instituational guideline aims for normocapnia in patients receiving HMV for COPD) and adequate patient recruitment (eg. stable patients without need for amendments to HMV treatment in the previous month).

Quality of life was different in different diagnostic groups in our study, which has been described before, making the SRI a valid diagnostic tool [19]. When comparing patients receiving ventilation through noninvasive or invasive interface, we found a small, but significant difference in overall quality of life to the favor of the noninvasive interface, mostly influenced by the subscales focusing on respiratory complaints, physical and social functions. This result is noteworthy despite the low ratio of invasively ventilated patients, and warrants further research of the subject. The viability of the Hungarian version of the SRI Questionnaire was similar to the previously translated versions, with 69.2% of patients able to self-administer the tool and a time of about 9 min to complete the questionnaire, which was identical to the SF-36 quality of life questionnaire widely used in Hungary. Ability to self administer the questionnaire, disease type and education level significantly inlfuenced time spent on the questionnaire, but the age of the patient was not an influencing faactor in our cohort. Missing items were less common with the SRI than the SF-36 questionnaire, attributing to a viable patient reported outcome tool.

Regarding validity, factor analysis explained 73.8% of the variation of the questionnaire, but it resulted in 13 scales, contributing in uneven fashion as verfified by scree plot. This is similar to results published by the Spanish and Portuguese validation of the SRI Questionnaire [7, 13] and has been explained by the initial methodology of creating the questionnaire scales, which was done by an expert panel rather than factor analysis [3]. Scale incongruencies have been described before with the SRI Questionnaire used in validation studies and are thought to be a result of limited study population numbers and differing patient composition compared to the original study [20, 21]. Despite exploratory factor analysis showing an increased number of scales in previously published validation studies, within scale incongruences tend to correlate well with each other, which was also true for our data. Despite the limited but more versatile patient population in Hungary, the Hungarian SRI Questionnaire’s validity proved to be similar to previously published translations with high overall Cronbach values.

Correlations between the Hungarian version of the SRI and SF-36 were verified in several corresponding scales, most notably in scales assessing physical function and well-being and in some extent social relation and social function. The summary score of the SRI Questionnaire and the general health score of the SF-36 questionnaire also showed reasonable correlation, verifying that the SRI Questionnaire was useful in assessing the overall quality of life in the patient group tested. The strength of the SRI Questinnaire for the HMV population is notable when studying the SRI-RC scale and it’s apparent low correlation with most of the SF-36 subscales, pointing out that respiratory complaints, an aspect especially crucial when assessing chronic respiratory failure patients, tends to be poorly reflected in scales of a general quality of life questionnaire, like the SF-36. This corraborates previous views that the SRI Questionnaire is better suited to evaluate important quality of life aspects in chronic respiratory failure.

Reliability of the Hungarian SRI Questionnaire was found to be high, with an overall Cronbach alpha value of 0.928 for the questionnaire, corresponding to strong internal integrity. Cronbach alpha values were less than 0.7 for three, the SRI-AS, −SR and -WB scales, notably ones we found more incongruent. Reproducibility was also sufficiently high for the questionnaire.

The limitations of our study include the smaller number of enrolled patients compared to those reported by some other validation studies, which can be explained by Hungary’s smaller overall population and reported low prevalence of patients treated with home mechanical ventilation, however its versatile patient group makes it a valid and valuable tool for the currently growing Hungarian practice as well as a new reference for newly recruiting HMV centers.

## Conclusions

In conclusion, the Hungarian version of the SRI Questionnaire is a viable, valid, reliable and reproduceable quality of life tool applicable for Hungarian patients treated with home mechanical ventilation and supplies additional information for the usefulness of the questionnaire in more versatile patient populations. Thus, further application of this version of the questionnaire for evaluation and monitoring of patients suffering from chronic respiratory failure is well founded.

## Supplementary information

**Additional file 1: Supplementary Material 1.** Hungarian SRI Questionnaire. The validated version of the Hungarian SRI Questionnaire (in Hungarian). For original German and validated English versions, see reference [3] and [8].

**Additional file 2: Supplementary Material 2.** Scoring Guide to the Hungarian SRI Questionnaire. The scoring guide to the validated version of the Hungarian SRI Questionnaire (in Hungarian). For original German and validated English versions, see reference [3] and [8].

**Additional file 3: Supplementary Material 3.** Comparison of international validation studies oft he SRI Questionnaire. The table contains patient numbers, Cronbach scores and factor analysis results of previously published validation studies.

## Data Availability

The Hungarian version of the SRI Questionnaire and the Scoring guide are included in this published article as supplementary information files (Suplementary files 1 and 2). The original German and validated English translation of these supplementary files are listed in the references [3, 8]. The datasets generated and analysed during the current validation study are available from the corresponding author on reasonable request.

## Abbreviations

ANOVA: Analysis of variance
BMI: Body mass index
COPD: Chronic obstructive pulmonary disease
EPAP: Expiratory positive airway pressure
FEV_1_: Forced expiratory volume in 1 s
FVC: Forced vital capacity
HMV: Home mechanical ventilation
ICS: Item correlation coefficient with its own scale
ICQ: Item correlation coefficient with the rest of the questionnaire
IPAP: Inspiratory positive airway pressure
NMD: Neuromuscular disease
OHS: Obesity hypoventilation syndrome
OSAS: Obstructive sleep apnea syndrome
PTB: Patient triggered breath
PEF: Peak expiratory flow
pCO2: Partial pressure of carbon dioxide
pO2: Partial pressure of oxygen
RCWD: Restrictive chest wall disease
SF-36: 36 Item Short Form Survey
SF36-PF: Physical functioning scale
SF36-PH: Role limitations due to physical health scale
SF36-EP: Role limitations due to emotional problems scale
SF36-E/F: Energy/fatigue scale
SF36-EWB: Emotional well-being scale
SF36-SF: Social functioning
SF36-P: Pain scale
SF36-GH: general health scale
SRI: Severe respiratory insufficiency questionnaire
SRI-RC: Respiratory complaints scale
SRI-PF: Physical functioning scale
SRI-AS: Attendant symptoms and sleep scale
SRI-SR: Social relationships scale
SRI-AX: Anxiety scale
SRI-WB: Psychosocial well-being scale
SRI-SF: Social functioning
SRI-SS: Summary score
Ti/Tt: Inspiratory-total time ratio
VTE: Expiratory tidal volume
