# Supplementary material for: Validation of the Hungarian version of the SRI Questionnaire
Source: BMC Pulm Med. 2020 May 7;20:130. doi: 10.1186/s12890-020-1171-5 (PMC7204221; doi:10.1186/s12890-020-1171-5)
Supplement: Supplementary file 1 — Additional file 1: Supplementary Material 1. Hungarian SRI Questionnaire. The validated version of the Hungarian SRI Questionnaire (in Hungarian). For original German and validated English versions, see reference [3] and [8]. [file 12890_2020_1171_MOESM1_ESM.pdf]

Severe Respiratory Insufficiency Questionnaire

SRI

Kérdőív

Súlyos Légzési Elégtelenségben szenvedő betegek közérzetének felmérésére

Tisztelt Betegünk!

Önt légzési zavarok miatt kezeljük intézményünkben. Jelenlegi közérzetének felméréséhez kérjük, töltse ki az alábbi kérdőívet! Kérjük, hogy minden kérdést válaszoljon meg a megfelelő válasz megjelölésével! A válaszadás természetesen önkéntes. Az összes adat az orvosi titoktartás részét képezi és szigorúan bizalmasan kezeljük. Amennyiben kérdése van, forduljon bizalommal osztályos orvosához!

Kódszám:

SRI

Ez a kérdőív az Ön általános közérzetére vonatkozik. Az alábbiakban kijelentéseket olvashat a mindennapi élet különböző jellemzőivel kapcsolatban.

Hogy érezte magát ***az elmúlt hét során?*** Kérjük, MINDEN kijelentésnél ahhoz a válaszhoz tegyen „X” jelet, amelyik a legjobban jellemzi az Ön állapotát!

	egyáltalán nem igaz - 2	kevéssé igaz - 1	részben igaz 0	meglehetősen igaz 1	teljesen igaz 2
1. Nehezemre esik felmenni a lépcsőn.	☐ - 2	☐ - 1	☐ 0	☐ 1	☐ 2
2. Evés közben nehezen kapok levegőt.	☐ - 2	☐ - 1	☐ 0	☐ 1	☐ 2
3. Esténként el tudok menni szórakozni.	☐ - 2	☐ - 1	☐ 0	☐ 1	☐ 2
4. Gyakran érzem magam rosszul.	☐ - 2	☐ - 1	☐ 0	☐ 1	☐ 2
5. Előfordul, hogy fizikai megerőltetés nélkül sem kapok levegőt.	☐ - 2	☐ - 1	☐ 0	☐ 1	☐ 2
6. Gyakran fáj a fejem.	☐ - 2	☐ - 1	☐ 0	☐ 1	☐ 2
7. Sok barátom és ismerősöm van.	☐ - 2	☐ - 1	☐ 0	☐ 1	☐ 2
8. Aggódom, hogy súlyosbodik a betegségem.	☐ - 2	☐ - 1	☐ 0	☐ 1	☐ 2
9. Könnyen elalszom.	☐ - 2	☐ - 1	☐ 0	☐ 1	☐ 2
10. Jól kijövök az emberekkel.	☐ - 2	☐ - 1	☐ 0	☐ 1	☐ 2
11. Néha szédülök.	☐ - 2	☐ - 1	☐ 0	☐ 1	☐ 2
12. Éjjel arra ébredek, hogy nem kapok levegőt.	☐ - 2	☐ - 1	☐ 0	☐ 1	☐ 2
13. Félek, hogy éjszaka nem kapok levegőt.	☐ - 2	☐ - 1	☐ 0	☐ 1	☐ 2
14. Gyakran fáj a nyakam.	☐ - 2	☐ - 1	☐ 0	☐ 1	☐ 2
15. Nagymértékben az otthonomhoz vagyok kötve.	☐ - 2	☐ - 1	☐ 0	☐ 1	☐ 2
16. Nehezemre esik a házimunka.	☐ - 2	☐ - 1	☐ 0	☐ 1	☐ 2

SRI

Hogy érezte magát ***az elmúlt hét során?*** Kérjük, MINDEN kijelentésnél ahhoz a válaszhoz tegyen „X” jelet, amelyik a legjobban jellemzi az Ön állapotát!

	egyáltalán nem igaz - 2	többnyire nem igaz - 1	részben igaz 0	többnyire igaz 1	teljes mértékben igaz 2
17. Éjszakánként gyakran felébredek.	☐ - 2	☐ - 1	☐ 0	☐ 1	☐ 2
18. Zavartalanul átalszom az éjszakát.	☐ - 2	☐ - 1	☐ 0	☐ 1	☐ 2
19. Gyakran nehezen veszem a levegőt.	☐ - 2	☐ - 1	☐ 0	☐ 1	☐ 2
20. Pozitívan látom a jövőt.	☐ - 2	☐ - 1	☐ 0	☐ 1	☐ 2
21. Egyedül érzem magam.	☐ - 2	☐ - 1	☐ 0	☐ 1	☐ 2
22. Beszéd közben nehezen kapok levegőt.	☐ - 2	☐ - 1	☐ 0	☐ 1	☐ 2
23. A látogatások nagyon kifárasztanak.	☐ - 2	☐ - 1	☐ 0	☐ 1	☐ 2
24. Sokat köhögök.	☐ - 2	☐ - 1	☐ 0	☐ 1	☐ 2
25. Légutaimban gyakran érzek váladékot.	☐ - 2	☐ - 1	☐ 0	☐ 1	☐ 2
26. Kerülöm azokat a helyzeteket, amelyek kínossá válhatnak számomra a légzési nehézségeim miatt.	☐ - 2	☐ - 1	☐ 0	☐ 1	☐ 2
27. Jól érzem magam a barátaim/ismerőseim körében.	☐ - 2	☐ - 1	☐ 0	☐ 1	☐ 2
28. Félek attól, hogy fulladásos roham tör rám.	☐ - 2	☐ - 1	☐ 0	☐ 1	☐ 2
29. Fizikai megerőltetés során nem kapok levegőt.	☐ - 2	☐ - 1	☐ 0	☐ 1	☐ 2
30. Idegesít, hogy a betegségem miatt korlátozva vagyok.	☐ - 2	☐ - 1	☐ 0	☐ 1	☐ 2
31. A házasságom/párkapcsolatom megsínyli a betegségemet.	☐ - 2	☐ - 1	☐ 0	☐ 1	☐ 2
32. El tudok menni bevásárolni.	☐ - 2	☐ - 1	☐ 0	☐ 1	☐ 2
33. Minden engem érdeklő szabadidős tevékenységben részt tudok venni.	☐ - 2	☐ - 1	☐ 0	☐ 1	☐ 2

SRI

Hogy érezte magát ***az elmúlt hét során?*** Kérjük, MINDEN kijelentésnél ahhoz a válaszhoz tegyen „X” jelet, amelyik a legjobban jellemzi az Ön állapotát!

	egyáltalán nem igaz - 2	többnyire nem igaz - 1	részben igaz 0	többnyire igaz 1	teljes mértékben igaz 2
34. Gyakran vagyok ingerült.	☐ - 2	☐ - 1	☐ 0	☐ 1	☐ 2
35. A betegségem miatt csak korlátozottan tarthatom a kapcsolatot barátaimmal/ismerőseimmel.	☐ - 2	☐ - 1	☐ 0	☐ 1	☐ 2
36. Boldogan élem az életem.	☐ - 2	☐ - 1	☐ 0	☐ 1	☐ 2
37. Részt tudok venni társasági eseményeken.	☐ - 2	☐ - 1	☐ 0	☐ 1	☐ 2
38. Gyakran vagyok szomorú.	☐ - 2	☐ - 1	☐ 0	☐ 1	☐ 2
39. A légzési panaszaim zavarnak a nyilvános helyzetekben.	☐ - 2	☐ - 1	☐ 0	☐ 1	☐ 2
40. Gyakran vagyok ideges.	☐ - 2	☐ - 1	☐ 0	☐ 1	☐ 2
41. Képes vagyok egyedül felöltözni.	☐ - 2	☐ - 1	☐ 0	☐ 1	☐ 2
42. Egész nap fáradt vagyok.	☐ - 2	☐ - 1	☐ 0	☐ 1	☐ 2
43. Elszigetelve érzem magam.	☐ - 2	☐ - 1	☐ 0	☐ 1	☐ 2
44. Jól boldogulok a betegségemmel.	☐ - 2	☐ - 1	☐ 0	☐ 1	☐ 2
45. Légzési panaszaim korlátoznak a mindennapi tevékenységek elvégzésében.	☐ - 2	☐ - 1	☐ 0	☐ 1	☐ 2
46. A betegségem megterheli a családi életemet.	☐ - 2	☐ - 1	☐ 0	☐ 1	☐ 2
47. Légzési panaszaim miatt meg kellett szakítanom a kapcsolatot más emberekkel.	☐ - 2	☐ - 1	☐ 0	☐ 1	☐ 2
48. Korlátozottak a lehetőségeim a szabadidő eltöltésére.	☐ - 2	☐ - 1	☐ 0	☐ 1	☐ 2
49. Általában elégedett vagyok az életemmel.	☐ - 2	☐ - 1	☐ 0	☐ 1	☐ 2

Köszönjük!
