# Supplementary material for: Validation of the Hungarian version of the SRI Questionnaire
Source: BMC Pulm Med. 2020 May 7;20:130. doi: 10.1186/s12890-020-1171-5 (PMC7204221; doi:10.1186/s12890-020-1171-5)
Supplement: Supplementary file 2 — Additional file 2: Supplementary Material 2. Scoring Guide to the Hungarian SRI Questionnaire. The scoring guide to the validated version of the Hungarian SRI Questionnaire (in Hungarian). For original German and validated English versions, see reference [3] and [8]. [file 12890_2020_1171_MOESM2_ESM.pdf]

Severe Respiratory Insufficiency Questionnaire

SRI

Kérdőív

Súlyos Légzési Elégtelenségben szenvedő
begek közérzetének felmérésére

Pontozási útmutató

Minden állításhoz határozzuk meg a hozzá tartozó értéket:

egyáltalán nem igaz	=>	1
kevéssé igaz	=>	2
részben igaz	=>	3
többnyire igaz	=>	4
teljesen igaz	=>	5

Az állítások többségénél az alábbi módon újrakódolt értéket kell meghatározni:

érték		újrakódolt érték
1	→	5
2	→	4
3	→	3
4	→	2
5	→	1

Újrakódolást igénylő állítások:

1, 2, 4, 5, 6, 8, 11, 12, 13, 14, 15, 16, 17, 19, 21, 22, 23, 24, 25, 26, 28, 29, 30, 31, 34, 35, 38, 39, 40, 42, 43, 45, 46, 47, 48.

Ezt követően az egyes skálák számítása következik az alábbiak szerint. Ehhez az adott skálához tartozó állítások legalább 50%-ának megválaszoltnak kell lenniük. Keressük meg a zárójelben felsorolt állításokat [a, b, c…..]. A kalkuláció eredménye egy 0 és 100 közötti szám, ahol a magasabb számok jobb életminőséget jeleznek.

Légzési panaszok

$$SRI - RC = \frac{\acute{A}tlag\ [2,5,12,19,22,24,25,29] - 1}{4} \bullet 100$$

Fizikális funkció

$$SRI - PF = \frac{\acute{A}tlag\ [1,16,32,33,41,45] - 1}{4} \bullet 100$$

Alvás- és ébrenlét tünetek

$$SRI - AS = \frac{\acute{A}tlag\ [6,9,11,14,17,18,42] - 1}{4} \bullet 100$$

Szociális kapcsolatok

$$SRI - SR = \frac{\acute{A}tlag\ [7,10,21,27,43,46] - 1}{4} \bullet 100$$

Szorongás

$$SRI - AX = \frac{\acute{A}tlag\ [8,13,26,28,39] - 1}{4} \bullet 100$$

Pszichés jólét

$$SRI - WB = \frac{\acute{A}tlag\ [4,20,30,34,36,38,40,44,49] - 1}{4} \bullet 100$$

Szociális viselkedés

$$SRI - SF = \frac{\acute{A}tlag\ [3,15,23,31,35,37,47,48] - 1}{4} \bullet 100$$

Összesített skála

Az összesített skála (*SRI-SS*) kiszámítása az alskálák (SRI-RC, SRI-PF, SRI-AS, SRI-SR, SRI-AX, SRI-WB, SRI-SF) értékeinek átlagolásával történik. Az SRI-SS nem számolható ki, ha bármelyik alskála hiányzik.
