# Supplementary material for: Validation of the Hungarian version of the SRI Questionnaire
Source: BMC Pulm Med. 2020 May 7;20:130. doi: 10.1186/s12890-020-1171-5 (PMC7204221; doi:10.1186/s12890-020-1171-5)
Supplement: Supplementary file 3 — Additional file 3: Supplementary Material 3. Comparison of international validation studies oft he SRI Questionnaire. The table contains patient numbers, Cronbach scores and factor analysis results of previously published validation studies. [file 12890_2020_1171_MOESM3_ESM.docx]

Supplementary Material 3.: Characteristics of previously published SRI Questionnaire translations

|  | **Number of patients recruited** | **Cronbach value of SRI-SS scale and (other scales)** | **Factor analysis: percentage of variance explained** | **Factor analysis: number of scales found** |
| --- | --- | --- | --- | --- |
| German (original)  (Windisch, Freidel et al. 2003) | 226 | 0.89  (0.73-0.89) | 59.8% | not done |
| Chinese  (Chen, Guan et al. 2017) | 149 | 0.95  (0.71-0.92) | 59.0% | 9 |
| English  (Ghosh, Rzehak et al. 2012) | 152 | 0.93  (0.77-0.89) | 70.0% | 10 |
| Japanese  (Oga, Taniguchi et al. 2017) | 56 | 0.92  (0.56-0.80) | not done | not done |
| Norwegian  (Markussen, Lehmann et al. 2015) | 127 | 0.94  (0.68-0.88) | not done | not done |
| Portuguese  (Ribeiro, Ferreira et al. 2017) | 93 | 0.84  (0.44-0.78) | 73.0% | 13 |
| Spanish  (Lopez-Campos, Failde et al. 2008) | 115 | 0.93  (0.63-0.85) | 60.0% | 13 |
| Hungarian | 104 | 0.93  (0.61-0.85) | 73.8% | 13 |

SRI-SS: Summary Scale of the Severe Respiratory Insufficiency Questionnaire
